# An Analysis of Two Hundred and Fifity Autopsies on Drunkards

**Published:** 1887-01

**Authors:** 


### *AN ANALYSIS OF TWO HUNDRED AND FIFTY AUTOPSIES ON DRUNKARDS.*

Illustrating the most prominent Anatomical Lesions of Chronic Alcoholism.

Dr. Formael, in a paper on the above subject, considered the most conspicuous lesions to be cyanotic induration of the kidneys, fatty infiltration of the liver, and mammillated stomach. His cases had been those in which there had been a history of a long-continued series of debauches, the subject often dying in one of these debauches, and did not include moderate drinkers or those who perished after imbibition of an enormous quantity of alcohol without any previous chronic excesses. He thought that the exposure, irregularities of diet, etc., incident to a state of drunkenness, had much—probably more than the alcohol itself—to do with the production of the lesions; but it was not at all possible to separate one from the other. He gave a long list of lesions considered by various authors to be results of chronic alcoholism, among which the cirrhotic liver with contraction held a prominent place. He had himself at one time considered cirrhosis a very frequent, if not almost necessary, concomitant of long-continued, excessive use of alcohol, and had even testified in court that a certain person was not likely to have been a hard drinker, because at the autopsy no cirrhosis of the liver was found. He had thought, too, that the connection between the two was so close that it was impossible to have a case of cirrhosis without a previous history of alcoholism, as is held by various authors. Therefore, it was surprising to him to meet in his two hundred and fifty autopsies with only six cases of cirrhosis of the liver with contraction. In two hundred and twenty cases, the liver was considerably or even very much enlarged, the enlargement in most cases proving to be due to a fatty infiltration. Cyanotic induration of the kidney and chronic gastritis, with mammillation of the stomach, were found in nearly every case. This cyanotic induration is peculiar, and differs from the cyanotic induration due to heart disease.—*Phil. Med. Times*, Dec. 12, 1886.

